# Electrochemical Hydrogen Peroxide Sensor Based on Macroporous Silicon

**DOI:** 10.3390/s18030716

**Published:** 2018-02-28

**Authors:** Naif H. Al-Hardan, Muhammad Azmi Abdul Hamid, Roslinda Shamsudin, Ensaf Mohammed AL-Khalqi, Lim Kar Keng, Naser M. Ahmed

**Affiliations:** 1 School of Applied Physics, Faculty of Science and Technology, Universiti Kebangsaan Malaysia (UKM), Bangi 43600, Malaysia; linda@ukm.edu.my (R.S.); p92953@siswa.ukm.edu.my (E.M.A.-K); karkeng.iamkklim@gmail.com (L.K.K.); 2 School of Physics, Universiti Sains Malaysia (USM), Penang 11800, Malaysia; naser@usm.my

**Keywords:** macroporous silicon, hydrogen peroxide, electrochemical sensors, amperometric sensors

## Abstract

Macroporous silicon was prepared through an anodization process; the prepared samples showed an average pore size ranging from 4 to 6 microns, and the depth of the pores in the silicon wafer was approximately 80 microns. The prepared samples were tested for hydrogen peroxide (H_2_O_2_) concentrations, which can be used for industrial and environmental sensing applications. The selected H_2_O_2_ concentration covered a wide range from 10 to 5000 μM. The tested samples showed a linear response through the tested H_2_O_2_ concentrations with a sensitivity of 0.55 μA μM^–1^∙cm^–2^ and lower detection limits of 4.35 μM at an operating voltage of 5 V. Furthermore, the electrode exhibited a rapid response with a response time of ca. two seconds. Furthermore, the prepared sensor showed a reasonable stability over a one-month time period.

## 1. Introduction

Macroporous silicon (MPS) has attracted the attention of several groups, owing to the well-known advantages of its large surface area and biocompatibility [1], as well as its showing promising behavior in several advanced applications, such as gas sensing [2,3], pH sensors [4], biochips [5], CMOS devices [6], drug delivery devices [1], biosensors [7,8], and in fuel cell devices [9] and waveguides [10]. Furthermore, it can be simply integrated with silicon technology processes [11]. Several published works have shown the excellent behavior for bio-sensing applications of porous silicon, which make it a promising candidate in the field of biosensors [11,12].

The important role of hydrogen peroxide (H_2_O_2_) in different areas, such as the food industry [13], manufacturing processes [14], and environmental protection [15] has been reported earlier. Furthermore, several neurodegenerative diseases, such as Parkinson’s and Alzheimer’s, are connected to excess amounts of H_2_O_2_ [16,17,18,19], and it also contributes to the formation of acid rain [20]. H_2_O_2_ has become one of the main substances in the monitoring of biological processes [21]. For these reasons, the determination of H_2_O_2_ levels has become a crucial task in various fields, including physiology, pathology, and the environment. Hence, sensitive and accurate determination of H_2_O_2_ in different environments is required. 

Various effective methods have been proposed and used to determine H_2_O_2_ concentrations, including titrimetric [22], spectrophotometry [23], chemi-luminescence, chromatography [24] and electrochemical methods [25,26]. However, most of these methods are bulky, use expensive [27] reagents, and require long analysis times. The electrochemical method has been in the spotlight for its cost-effective characteristics, low detection limit, high sensitivity, fast response, and excellent selectivity [27,28]. Most prepared electrodes under test that have been used for the electrochemical method are modified by immobilization with catalytic substances that show better sensitivity and selectivity [25,29,30]. Unfortunately, these types of electrodes face some drawbacks as well, such as high cost, instability, and inability to work effectively under proper conditions [28]. 

Previously, we reported on the application of zinc oxide (ZnO) nanorods for H_2_O_2_ detection [31], the prepared ZnO nanorods showed a linear response in the range of 10 μM to 700 μM with a sensitivity of 295 nA μM^–1^∙cm^–2^. The low-range response may be due to the degradability of ZnO once it is in contact with the analyte [32,33], or to the limit in the active sites. On the other hand, porous silicon has shown more stable performance in several biosensing applications. Here, we report on the application of a non-enzymatic H_2_O_2_ sensor based on MPS for environmental and industrial applications. The electrode is tested in H_2_O_2_ dissolved in deionized water covering the range between 10 μM and 5000 μM.

## 2. Materials and Methods 

The macroporous silicon (MPS) samples used in this study were fabricated using an anodization processor. The processor was explained in detail in our previous published work [4]. In summary, an n-type silicon wafer (Si) with (100) orientation and resistivity value of ~1–10 Ω∙cm was cut into four pieces. The samples were then cleaned via the RCA (Radio Corporation of America) method. Then, the samples were inserted into a homemade Teflon electrochemical cell. The polished side of the Si sample faces the cathode electrode (Pt). The cell is filled with a mixture of hydrofluoric acid (HF 48%) and ethanol (C_2_H_5_OH 98%) at ratio of 1:4 *v*/*v*. The system was then supplied with a constant current density of 30 mA/cm^2^ for 15 min under illumination from a 100 W tungsten lamp. The fabricated MPS samples were then rinsed with deionized water and dried with a nitrogen gas flow.

The surface morphology and cross-section details of the fabricated MPS were investigated via field emission scanning electron microscopy (FE-SEM) Zeiss SUPRA 55-VP (Carl Zeiss AG, Oberkochen, Germany).

From the prepared MPS samples, small pieces were scribed and mounted on a copper strip from a printed circuit board (PCB). Conductive wire was connected between the Si wafer and the copper strip using a conductive paste. Epoxy resin was employed to encapsulate the MPS and the copper line of the PCB. The prepared electrodes were left to cure for at least 24 h at room temperature. An area of approximately 0.55 cm × 0.35 cm free of epoxy was used as a sensing window. 

The sensing characteristics of the fabricated MPS electrode toward different concentrations of H_2_O_2_ were tested by employing the 6517A electrometer (Keithley Instruments, Inc., Cleveland, OH, USA), attached to a host personal computer via GPIB-USB cable. LabView software was utilized to control the electrometer and save the result data for further analysis. The manufactured MPS electrode was considered as the working-electrode, and a platinum wire (diameter of 0.5 mm) as the counter-electrode. The measured current in the current-voltage (I–V) test was obtained in the voltage range between 0 and 6 V. The measurements were repeated at least twice for each step, and the average of the measurements was calculated.

The H_2_O_2_ stock solution was prepared by mixing a given amount of H_2_O_2_ (30 wt. % in H_2_O) from Sigma-Aldrich in double-deionized water (DIW). Then the prepared stock was diluted to the desired concentrations. The concentration of the H_2_O_2_ used to test the manufactured MPS electrode was in the range of 10 μM to 5000 μM. The measurements were performed under ambient atmospheric conditions (23 °C, and 50% relative humidity).

## 3. Results

**Figure 1:**
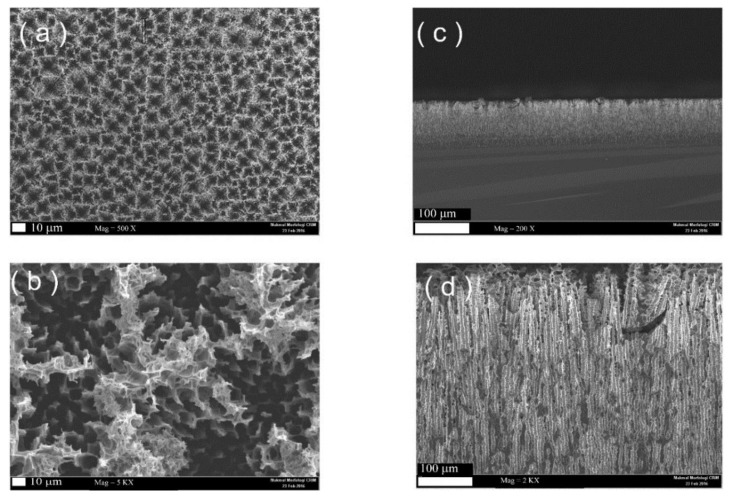
The FE-SEM image of the prepared MPS surface: (**a**) the surface; (**b**) higher magnification of the MPS surface; (**c**) the cross-section of the prepared MPS; and with higher magnification (**d**).

Initially, a visual inspection at the surface of the fabricated MPS shows the formation of a smooth light brown color membrane on the Si wafer surface, which confirms the formation of the pores. The micro image of the fabricated MPS sample is depicted in Figure 1. The nearly uniform distribution of the formed pores on the Si surface is clear from the image (Figure 1a). The average pore size was in the range of 4–6 microns. On the other hand, a cross-section image of the same fabricated sample reveals that the depth of the fabricated MPS was approximately 80 microns (Figure 1c), which makes it a suitable candidate for amperometry-based electrochemical sensors [11]. In addition, Figure 1b,d depicts a larger magnification of the above images, it also shows the large surface area of the pores, which result in a higher exposed surface area of the MPS with the analyte.

**Figure 2:**
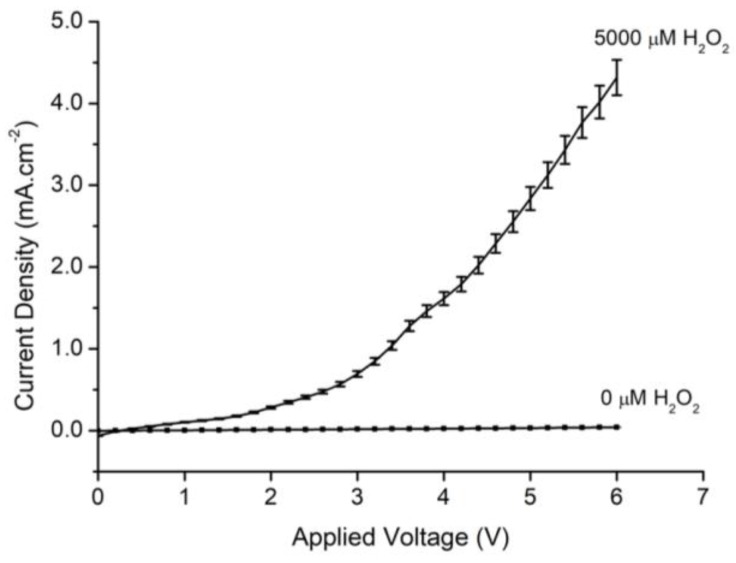
The I–V characterization of MPS versus Pt electrodes with 5000 μM H_2_O_2_ and pure deionized water.

To investigate the sensing ability of the fabricated MPS with respect to different H_2_O_2_ concentrations, the current was measured versus H_2_O_2_ at a concentration of 5000 μM. Figure 2 presents the I–V behavior of the fabricated MPS electrode. The figure reveals the difference between the measured current density in the DIW and 5000 μM H_2_O_2_ stock solution. It is obvious that the current density is drastically increased as the environment changes from the DIW to the H_2_O_2_ solution. This increase in the current density implies the excellent electro-catalytic activity of the fabricated MPS electrode for the oxidation of H_2_O_2_ diluted in DIW. Furthermore, it proves the modification in the electrical properties of the fabricated MPS electrode with the introduction of H_2_O_2_ molecules.

**Figure 3:**
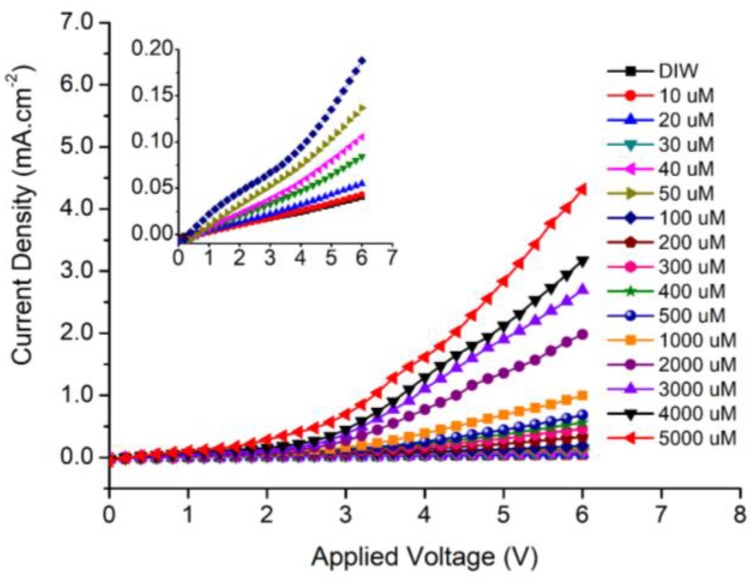
The current density versus the applied voltage with different H_2_O_2_ concentrations. The inset figure shows the details of low H_2_O_2_ concentrations (DIW to 100) μM.

For more details on the I–V behavior of the manufactured MPS electrode, the electrode was examined with different H_2_O_2_ amounts. The stock solution was diluted in the range from 5000 μM to 10 μM, and the result currents were recorded. Figure 3 depicts the I–V characteristics, and the gradual increase in the current density as the H_2_O_2_ concentrations is increased is noteworthy. The inset shows the measured current density at low H_2_O_2_ concentrations from DIW to 100 μM.

It is known that the oxygen molecules [34,35] dissolved in water are adsorbed (chemisorbed) on the MPS active sites; consequently, it converts to active oxygen ions with the capture of electrons from the surface of MPS. This can be understood through the following reactions [31,35]:

(1) $$\begin{array}{l} O_{2}\;(dissolved)\to O_{2}\;(adsorbed)\\ O_{2}\;(adsorbed)+e^{-}\to O_{2}^{-}\;(adsorbed) \end{array}$$

Once the H_2_O_2_ is added to the water, the active oxygen ions will react with the analyte. As a result, this will generate free electrons, as can be seen in the following reaction:

(2) $$2H_{2}O_{2}+O_{2}^{-}(adsorbed)\to 2H_{2}O+2O_{2}+2e^{-}$$

Subsequently, the measured current will increase with the addition of more H_2_O_2_, which results in the generation of more free charge carriers. 

**Figure 4:**
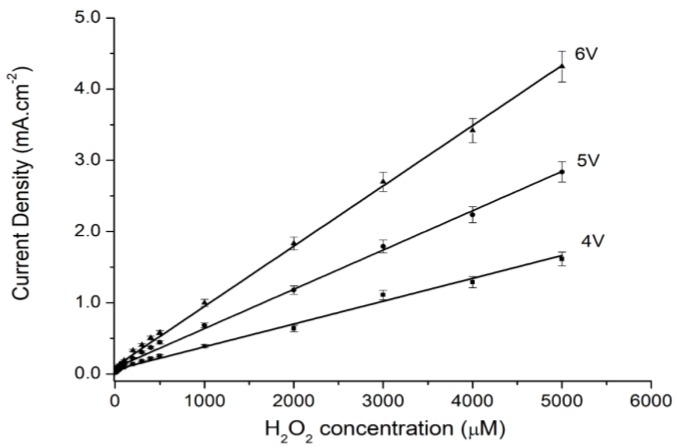
The current density versus the applied voltage with different H_2_O_2_ concentrations.

The effect of applied voltage at 4 V, 5 V, and 6 V on the current values measured at various amounts of H_2_O_2_ can be seen in Figure 4. The increase of the current with the applied voltage is noticeable. 

From the gradients of each applied voltage versus H_2_O_2_ concentrations at each applied voltage, the sensitivity of the MPS for H_2_O_2_ can be calculated. In Table 1, the results of the sensitivity and the lower detection limits (LODs) of the fabricated MPS electrodes are tabulated. The tabulated results reveal that with an increase in the applied voltage from 4 V to 6 V, the sensitivity increased from 0.32 to 0.85 Μa μM^–1^∙cm^–2^. On the other hand, the LOD shows an insignificant change in value at the same operating voltage.

**Figure 5:**
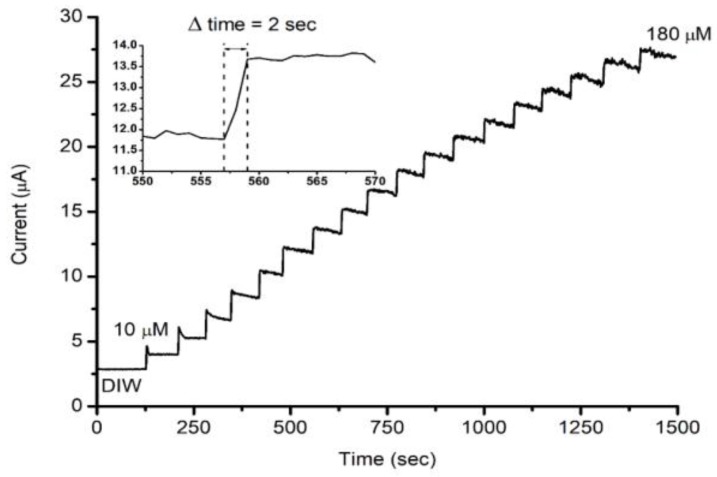
The current response versus time at an applied voltage of 5 V with accumulated concentrations of H_2_O_2_, each step is 10 μM. The inset shows the response time of approximately 2 s at each step.

The dynamic current-time (I-t) behavior was also tested for the manufactured electrode. The stock solution was diluted so that each drop of the diluted sample was equivalent to 10 μM in a 20 mL DIW cell. The applied voltage was fixed at 5 V, and the solution was continuously stirred. Figure 5 reveals the I-t behavior of the manufactured MPS electrode, the rapid response at each drop of H_2_O_2_ is noticeable, with a response time of approximately two seconds.

**Figure 6:**
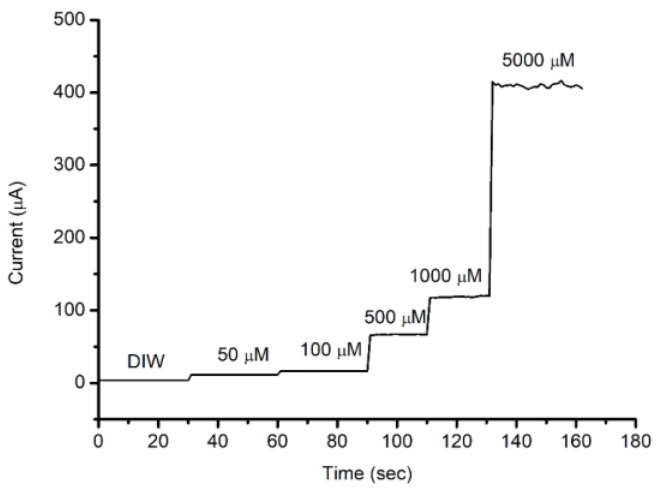
The current response versus time at an applied voltage of 5 V with concentrations of H_2_O_2_ in the range of 0 to 5000 μM.

Figure 6, on the other hand, reveals the response of the manufactured MPS electrode at higher H_2_O_2_ concentrations. The electrode shows a stable current output as the H_2_O_2_ concentration increases to 5000 μM.

The manufactured MPS electrode shows a promising behavior for sensor work in industrial and environmental applications for the detection of H_2_O_2_ amounts. In Table 2, several materials are shown for comparison with the results obtained in this study. The results in this work were within the range of different materials and compounds tested for H_2_O_2_. Despite there being many materials used to test H_2_O_2_ concentrations, most of those materials are expensive or have time-consuming preparation processes. This make MPS a good competitive material on account of its simple preparation and its compatibility with IC process technology.

**Figure 7:**
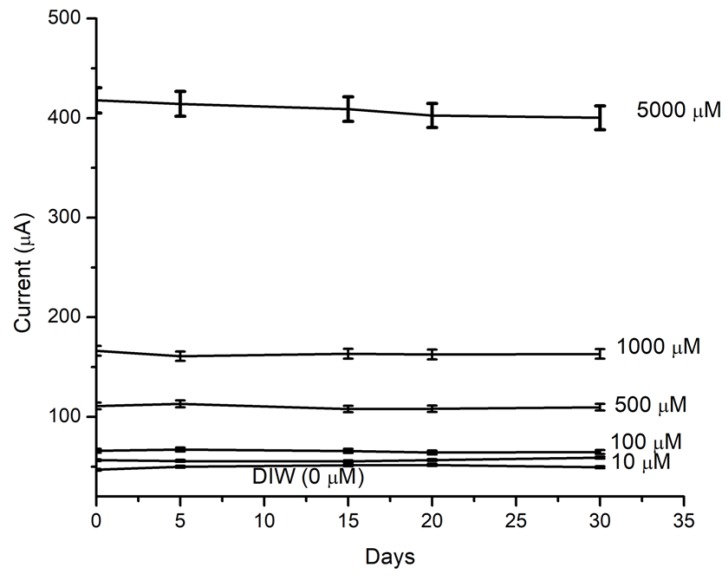
The stability of the measured current for one month at an applied voltage of 5 V. The concentrations of H_2_O_2_ were 0, 10, 100, 500, 1000, and 5000 μM.

In addition, the long-term stability of the manufactured MPS was tested at specific H_2_O_2_ concentrations. The results are shown in Figure 7, which shows a stable behavior for a period time of one month.

## 4. Conclusions

In summary, we report the application of MPS for H_2_O_2_ sensing. The manufactured sensor shows excellent behavior in terms of its linearity within a wide concentration range of the analyte (10 to 5000 μM), sensitivity (0.55 μA μM^–1^∙cm^–2^), and LOD (4.35 μM), with a fast response time of two seconds. Furthermore, the manufactured electrode shows a stable behavior over a time period of one month. 

## Figures and Tables

**Table 1:** Depicts the sensitivity and LOD with the applied voltage values.

Applied Voltage (V)	Sensitivity (μA μM^−1^·cm^−2^)	LOD (μM) (SNR = 3)
4	0.32 ± 0.082	4.38
5	0.55 ± 0.018	4.35
6	0.85 ± 0.023	3.61

**Table 2:** Some materials used for H_2_O_2_ detection as compared to this study.

Electrode	Detection Limit (μM)	Linea Range (μM)	References
Pt/TeO_2_-NWs	0.60	2–16,000	[26]
CdOx in EIS structure	1	1–200	[36]
Co_3_O_4_ NW	2.4	15–675	[37]
Pt NP	1.23	5–2000	[38]
Ag NW	29.2	100–3100	[39]
MoS2 NP	0.002	5–100	[40]
CuO/Cu foil	16.7	42.5–40,000	[41]
Pt/TiO_2_/single-walled carbon nanotube	0.73	0–3500	[42]
Cu porous Si	0.27	500–3780	[43]
MPS/copper PCB	4.35	10–5000	This study
